# Targeted Long-Read Bisulfite Sequencing Identifies Differences in the *TERT* Promoter Methylation Profiles between *TERT* Wild-Type and *TERT* Mutant Cancer Cells

**DOI:** 10.3390/cancers14164018

**Published:** 2022-08-19

**Authors:** Seungjae Lee, Ti-Cheng Chang, Patrick Schreiner, Yiping Fan, Neeraj Agarwal, Charles Owens, Reinhard Dummer, John M. Kirkwood, Raymond L. Barnhill, Dan Theodorescu, Gang Wu, Armita Bahrami

**Affiliations:** 1 Department of Pathology, St. Jude Children’s Research Hospital, Memphis, TN 38105, USA; 2 Center for Applied Bioinformatics, St. Jude Children’s Research Hospital, Memphis, TN 38015, USA; 3 Cedars-Sinai Samuel Oschin Comprehensive Cancer Institute, Los Angeles, CA 90048, USA; 4 Department of Surgery, University of Colorado-Anschutz Medical Campus, Aurora, CO 80045, USA; 5 Department of Dermatology, University Hospital Zurich, 8091 Zurich, Switzerland; 6 Department of Pathology, University of Pittsburgh Cancer Center, Pittsburgh, PA 15232, USA; 7 Department of Translational Research, Institut Curie, 75248 Paris, France; 8 Department of Surgery (Urology), Cedars-Sinai Medical Center, Los Angeles, CA 90048, USA; 9 Department of Pathology and Laboratory Medicine, Emory University School of Medicine, Atlanta, GA 30307, USA

**Keywords:** *TERT* promoter, methylation, bisulfite sequencing, allele-specific methylation, proximal and distal promoters

## Abstract

**Simple Summary:**

*TERT* promoter methylation is enriched in cancers lacking *TERT* genetic alterations (wild-type cancers), but its functional impact on TERT transcription remains elusive. We developed a long-read bisulfite-sequencing platform to characterize the *TERT* promoter methylation profile at a single-molecule level. In wild-type cancer cell lines, both epialleles were hypermethylated symmetrically on the *TERT* distal promoter. In the core and proximal promoter, by contrast, the transcribed epialleles were significantly more hypomethylated than the silent epialleles. Decitabine-therapy reduced the core and proximal (not the distal) promoter methylation and reactivated the silent allele. We showed that *TERT* allele-specific expression is amenable to in vitro epigenetic manipulation in wild-type cancers.

**Abstract:**

Background: *TERT* promoter methylation, located several hundred base pairs upstream of the transcriptional start site, is cancer specific and correlates with increased TERT mRNA expression and poorer patient outcome. Promoter methylation, however, is not mutually exclusive to *TERT* activating genetic alterations, as predicted for functionally redundant mechanisms. To annotate the altered patterns of *TERT* promoter methylation and their relationship with gene expression, we applied a Pacific Biosciences-based, long-read, bisulfite-sequencing technology and compared the differences in the methylation marks between wild-type and mutant cancers in an allele-specific manner. Results: We cataloged *TERT* genetic alterations (i.e., promoter point mutations or structural variations), allele-specific promoter methylation patterns, and allele-specific expression levels in a cohort of 54 cancer cell lines. In heterozygous mutant cell lines, the mutant alleles were significantly less methylated than their silent, mutation-free alleles (*p* < 0.05). In wild-type cell lines, by contrast, both epialleles were equally methylated to high levels at the *TERT* distal promoter, but differentially methylated in the proximal regions. ChIP analysis showed that epialleles with the hypomethylated proximal and core promoter were enriched in the active histone mark H3K4me2/3, whereas epialleles that were methylated in those regions were enriched in the repressive histone mark H3K27me3. Decitabine therapy induced biallelic expression in the wild-type cancer cells, whereas the mutant cell lines were unaffected. Conclusions: Long-read bisulfite sequencing analysis revealed differences in the methylation profiles and responses to demethylating agents between *TERT* wild-type and genetically altered cancer cell lines. The causal relation between *TERT* promoter methylation and gene expression remains to be established.

## 1. Introduction

*Telomerase reverse transcriptase (TERT)* encodes the catalytic subunit of telomerase, the enzyme required by most proliferative cells to counteract telomere shortening during cell divisions [1]. Aggressive cancers maintain telomere integrity usually by expressing TERT mRNA, although by various means. The *TERT* promoter is commonly genetically altered and/or methylated in cancer [2]. *TERT* genetic alterations include heterozygous cis-activating *TERT* promoter point mutations—the most frequent non-coding mutation in cancer—[3,4,5,6] and *TERT* structural variations (i.e., rearrangements, amplifications) [2,7,8]. These genetic alterations are mutually exclusive to each other and to the telomerase-independent Alternative Lengthening of Telomeres (ALT) mechanism [2,6,7,8,9], consistent with their functional redundancy in cancer. Cancer cell lines with a heterozygous *TERT* genetic alteration only express the genetically altered allele [5,8,10,11,12].

Many other cancers express TERT mRNA by less clearly defined means that do not involve *TERT* genetic alterations (i.e., wild-type cancers) [5,13,14,15,16,17]. Wild-type cancers commonly display an altered pattern of *TERT* promoter DNA hypermethylation [15,18,19]. *TERT* promoter hypermethylation occurs at CpG dinucleotides located several hundred base pairs (bp) upstream of the transcriptional start site (TSS) [20]. This pattern of *TERT* promoter hypermethylation is cancer specific and significantly correlates with increased TERT expression and poorer patient outcome [2,20,21]. Although *TERT* promoter hypermethylation is enriched in wild-type cancers, it is not mutually exclusive to *TERT* activating genetic alterations, as predicted for functionally redundant mechanisms [2,21,22]. In fact, studies in mutant cancer cell lines have shown that the transcriptionally active mutant allele is less methylated than its transcriptionally silent mutation-free counterpart [23,24].

Cancer cell lines with the wild-type *TERT* express either one or both alleles, on a case-by-case basis [5]. However the underlying reason as to why some wild-type samples have bi-allelic while others have mono-allelic TERT expression is not known [25]. Additionally, the relationship between the altered patterns of DNA methylation and gene expression has not been comprehensively studied at the allele-specific level. Furthermore, these altered patterns have not been systemically compared between wild-type and mutant cancers.

In this study, we cataloged *TERT* genetic alterations (promoter mutations or structural rearrangements), allele-specific promoter methylation patterns, and allele-specific expression levels in 70 metastatic melanomas and 54 cancer cell lines. We used a long-read sequencing technique to annotate methylation patterns in consecutive CpG sites at the single molecule level. In a subset of the cell lines, we also examined how decitabine treatment, aimed at removing DNA methylation marks, affected *TERT* promoter methylation and gene expression per allele. Our results revealed previously unappreciated differences in the epigenetic modification and gene expression between wild-type and genetically altered cancer cell lines.

## 2. Materials and Methods

### 2.1. Clinical Samples

The samples consisted of formalin-fixed paraffin-embedded (FFPE) metastatic melanomas (Supplemental Table S1) from 70 patients (36 females; 34 males), ranging in age from 16 to 93 years old (median, 59). The histologic subtypes of primary tumors were non-acral cutaneous (*n* = 50, including 13 nodular; 15 superficial spreading; 1 lentigo malignant melanoma; 21 not otherwise specified), acral (*n* = 11), mucosal (*n* = 6), and unspecified (*n* = 3).

### 2.2. Cell Lines

The 54 cancer cell lines used in this study consisted of 11 wild-type (4 urothelial cancer, 5 melanoma, and 2 lung cancer), 2 with structural rearrangement (1 melanoma, 1 glioblastoma), and 41 mutant (20 melanoma and 21 urothelial cancer) cell lines (Supplemental Table S1). WM-39, WM-46, WM-51, WM-88, and WM-4002 (Rockland Antibodies and Assays; Gilbertsville, PA, USA) were grown in Tu2% media (80% MCDB-153 (Sigma-Aldrich; St. Louis, MO, USA), 20% Leibovitz’s L-15 (ATCC; Manassas, VA, USA), 5 µg/mL bovine insulin (Sigma-Aldrich; St. Louis, MO, USA), 1.68 mM CaCl2 (Sigma-Aldrich; St. Louis, MO, USA), and 2% FBS (Peak Serum; Wellington, CO, USA)). NCI-H358 and NCI-H2122 cells (ATCC; Manassas, VA, USA) were grown in RPMI-1640 (ThermoFisher; Waltham, MA, USA) with 10% FBS. LN-18 cells (ATCC; Manassas, VA, USA) were grown in DMEM (ThermoFisher; Waltham, MA, USA) with 5% FBS. 253J cells (MD Anderson) were grown in MEM, 0.1 mM non-essential amino acids (ThermoFisher; Waltham, MA, USA), 1 mM sodium pyruvate (ThermoFisher; Waltham, MA, USA) with 10% FBS. UMUC3 cells (ATCC; Manassas, VA, USA) were grown in MEM (ThermoFisher; Waltham, MA, USA), 1 mM sodium pyruvate, and 10% FBS. VMCUB3 cells were grown in MEM, 0.1 mM non-essential amino acids, and 1 mM sodium pyruvate with 10% FBS.

### 2.3. TERT Promoter Sanger Sequencing

Genomic DNA (gDNA) was isolated from FFPE tissue samples or cell lines with Maxwell 16 LEV DNA kit (Promega; Madison, WI, USA) or DNeasy Blood and Tissue Kit (Qiagen; Hilden, Germany). *TERT* promoter Sanger sequencing was performed as described previously [22].

### 2.4. Fluorescence In Situ Hybridization

*TERT* rearrangements in clinical samples and in some cell lines (Supplemental Figure S1) were determined by fluorescence in situ hybridization (FISH). Cell lines were prepared into cell blocks. Briefly, 10 million cells were collected and centrifuged at 1000 RPM for 15 min into a cell pellet, fixed in 10% NBF for a minimum of 6 h, spun in a centrifuge at 1000 RPM for 15 min, suspended in PBS, and spun in a centrifuge at 1000 RPM for 15 min and processed in histology laboratory for HistoGel-based cell block preparation. Interphase break-apart FISH was performed on 4 μm FFPE tissue or cell block sections as previously described [22]. *TERT* rearrangements were determined by break-apart FISH using bacterial artificial chromosome (BAC) CH17-75N21- and CH17-410B01-templated probes for *TERT*. The Cy3-labeled TelG probe (PNAbio, Newbury Park, CA, USA) was used for telomeric DNA FISH as described previously [26].

### 2.5. TERT mRNA Expression

FFPE sample RNA was isolated using the Maxwell 16 LEV RNA kit (Promega; Madison, WI) and cDNA was prepared with Vilo SuperScript (ThermoFisher; Waltham, MA). TERT mRNA expression levels were measured by qRT-PCR, as previously described [22]. Spitz tumor and normal skin samples were used as negative controls for *TERT* alterations and expression (Supplemental Table S1).

### 2.6. High-Throughput TERT Promoter Methylation Analysis

*TERT* promoter methylation status in FFPE clinical specimens was determined by gDNA bisulfite-treatment before PCR amplification, followed by high throughput amplicon sequencing. *TERT* promoter methylation status from 425 to 610 bp upstream of the TSS (chr5:1,295,586–1,295,771; hg19) was analyzed from FFPE gDNA as described previously [15].

The *TERT* promoter (extending 663 bp upstream of the TSS, chr5:1,295,135–1,295,824; hg19) was amplified from ~500 ng of bisulfite-treated cell line gDNA (EZ DNA Methylation Gold kit, Zymo Research; Irvine, CA, USA) using barcoded forward (5′-GGGTTAGGGTTTTTTA-3′) and reverse primers (5′-CCRCCTAAAAACCTACAAAAAAAAATAAC-3′). A total of 600 µL of pooled sample PCR amplicons were cleaned with 700 µL of PB beads (Pacific Biosciences; Menlo Park, CA, USA) and eluted in 30 µL of 10 mM Tris-HCl, pH 8.5, as per the manufacturer’s instructions. Data was collected on a Pacbio Sequel instrument (University of Arizona Genomics Institute). Smrttools (version 5.0.1, PACBIO, Menlo Park, CA, USA) were used for preprocessing the Pacbio reads. Specifically, the reads were demultiplexed by lima and circular consensus (CCS) reads were generated using the ccs tool with parameters of “—maxLength 2000—minPasses 3—minPredictedAccuracy 0.95”. The CCS reads were aligned against the human reference genome (hg19) to call methylation status using Bismark [27].

The methylation profiles and mutation status of the *TERT* promoter (chr5:1,293,900–1,296,000) were analyzed by in-house workflows. One urothelial cancer (TCCSUP) and 3 melanoma (WM-3670, WM-3755, and WM-1158) mutant cell lines that were completely unmethylated throughout the amplicon were excluded from this analysis because the mechanism of promoter methylation is likely not involved in transcription in these cases (Supplemental Table S1). We analyzed the methylation profiles for 3 distinct promoter regions in this study: the core promoter (187 bp, chr5:1,295,135–1,295,321), the proximal promoter (264 bp, chr5: 1,295,322–1,295,585), and the frequently methylated distal promoter (239 bp, chr5: 1,295,586–1,295,824) regions. The reads of each cell line were iteratively clustered based on the breadth and depth of methylation level until the optimized number of read groups were identified (maximum allowed group number 4) [28]. For cell lines with two predominant read groups, the groups were assigned as low- and high-methylated epialleles. Sanger sequencing, ddPCR, and high-throughput sequencing verified promoter mutation status (genomic position, hg19: 1,295,161, 1,295,228, 1,295,242, 1,295,243, and 1,295,250) [15].

### 2.7. Chromatin Immunoprecipitation (ChIP) and Bisulfite PCR-Sequencing

H3K4me2/3 and H3K27me3 ChIP were performed on 3 wild-type *TERT* (NCI-H2122, 253J, and NCI-H358) and 2 mutant *TERT* cancer cell lines (UMUC3 and A375) according to described protocols [10,24] with some modifications. Briefly, 5 million cells were cross-linked with 1% Formaldehyde (ThermoFisher; Waltham, MA, USA) and chromatin was eluted with ChIP lysis buffer High Salt (Santa Cruz; Dallas, TX, USA) and sheared to 200–500 bp using Bioruptor (Diagenode; Denville, NJ, USA). Sheared chromatin was diluted by ChIP Dilution Buffer-II (BOSTON BIOProducts; Ashland, MA, USA) and immunoprecipitated with antibody for H3K4me2/3 (Abcam; Cambridge, UK) and H3K27me3 (EMD Millipore; Burlington, MA, USA) to target active and repressive histone marks, respectively, and incubated overnight at 4 °C. Protein A- or G-coated magnetic beads (Diagenode; Denville, NJ, USA) were used to capture the antibodies for 2 h at 4 °C. IPure kit v2 (Diagenode; Denville, NJ, USA) was used to recover DNA after ChIP or collected input DNA. Antibodies against normal rabbit (EMD Millipore; Burlington, MA, USA) and normal mouse IgG (EMD Millipore; Burlington, MA, USA) were used for negative controls. A human ChIP-seq grade GAPDH TSS primer pair (Diagenode; Denville, NJ, USA) was used for ChIP validation. The relative enrichment of H3K4me2/3 and H3K27me3 at the *TERT* promoter was determined by Sanger sequencing and the *TERT* promoter mutation digital droplet PCR (ddPCR; RainDance; Lexington, MA, USA) assays using recommended protocols (ThermoFisher; Waltham, MA, USA).

To determine *TERT* promoter methylation patterns associated with active or silent histone marks in these cell lines, ChIP DNA was bisulfite converted using an EZ DNA Methylation-Gold Kit (Zymo Research) and PCR amplified for 45 cycles at 95 °C for 15 s, 63 °C for 20 s, and 68 °C for 40 s using primers spanning the region from 1,295,224 to 1,295,546 of the *TERT* promoter with 5′-GGAAAGGAAGGGGAGGGGTTGGGAG-3′ and 5′-CCCCTCCCTCRAATTACCCCACAACCTAAAC-3′. Amplicons were purified and used for high-throughput sequencing (PACBIO) or Sanger sequencing.

*TERT* allele-specific expression in cell lines was measured in rs2736098 or rs2853690 (ThermoFisher; Waltham, MA, USA) heterozygotes using ddPCR TaqMan SNP assays compatible with cDNA of spliced mRNA. Heterozygous sample gDNA allele ratios were quantified by ddPCR [29]. Allele-specific expression was then calculated as follows:

allele1 or 2 gDNA ratio = (allele1 or 2 gDNA droplet counts)/(total gDNA droplet counts)
allele1 or 2 cDNA ratio = (allele1 or 2 cDNA droplet counts)/(total cDNA droplet counts)
normalized allele1 or 2 cDNA ratio = (allele1 or 2 cDNA ratio)/(allele1 or 2 gDNA ratio)
allele-specific expression1 or 2 = (normalized allele1 or 2 cDNA ratio)/(normalized allele1 cDNA ratio + normalized allele2 cDNA ratio)

Samples with mono-allelic expression had ≥9:1 allele-specific expression ratio [5,30,31] as measured by at least 10 positive signal droplets [31,32]. Thermocycler conditions for all TaqMan SNP assays were as recommended by the manufacturer. Data was analyzed using RainDrop Analyst II software (BioRad; Hercules, CA, USA).

### 2.8. Cell Line Decitabine Treatment

Effects of decitabine (Sigma-Aldrich; St. Louis, MO, USA) on allele-specific expression and allele-specific methylation were measured in wild-type (*n* = 4), mutant (*n* = 5), and *TERT* rearranged (*n* = 2) cell lines with methylated promoters and heterozygous for an assayable exonic SNP. Different drug concentrations and treatment lengths were tested to find the condition of half maximal inhibitory cell line growth. Cell line allele-specific expression measurements were then made using drug concentrations 10-fold lower and 10-fold higher than this condition. LN18, WM-51, and VMCUB3 cell lines were treated with either 0.3 or 30 µM decitabine, the NCI-H2122 cell line was treated with either 0.1 or 3 µM decitabine, the NCI-H358 cell line was treated with either 0.01 or 30 µM decitabine, UMUC3 and WM-46 cell lines were treated with either 0.03 or 3 µM decitabine, WM-4002 and 253J cell lines were treated with either 0.01 or 1 µM decitabine, and the WM-88 cell line was treated with 0.3 µM decitabine for 3–7 days (Supplemental Tables S3 and S5). Cell viability was measured with CellTiter-Glo Viability Assay (Promega; Madison, WI, USA). Media was replaced daily with freshly diluted DMSO or drug. Genomic DNA and RNA were extracted from treated cells at different time points using Blood and Tissue DNA Isolation and RNeasy Plus Micro kits (Qiagen; Hilden, Germany), respectively. cDNA was prepared with Vilo SuperScript. cDNA prepared without reverse transcriptase served as another negative control for some allele-specific expression measurements.

## 3. Results

### 3.1. TERT Promoter Mutation and TERT Distal Promoter Methylation Are Not Mutually Exclusive at the Clinical Sample Level in Melanoma

We first sought to determine if *TERT* promoter genetic alterations (i.e., promoter mutations or structural rearrangements) and the cancer-associated (distal) promoter methylation are mutually exclusive in clinical cancer specimens. To answer this question, we selected formalin-fixed paraffin-embedded (FFPE) samples of malignant melanoma, a cancer type enriched in both promoter mutations and methylation [2,22]. We segregated 70 clinically established metastatic melanoma specimens into wild-type and genetically altered groups by Sanger sequencing and fluorescence in situ hybridization (FISH). Next, we measured the *TERT* distal promoter methylation levels in each specimen by high-throughput targeted bisulfite amplicon sequencing. Finally, we compared the relative methylation levels between wild-type and genetically altered categories.

**Figure 1:**
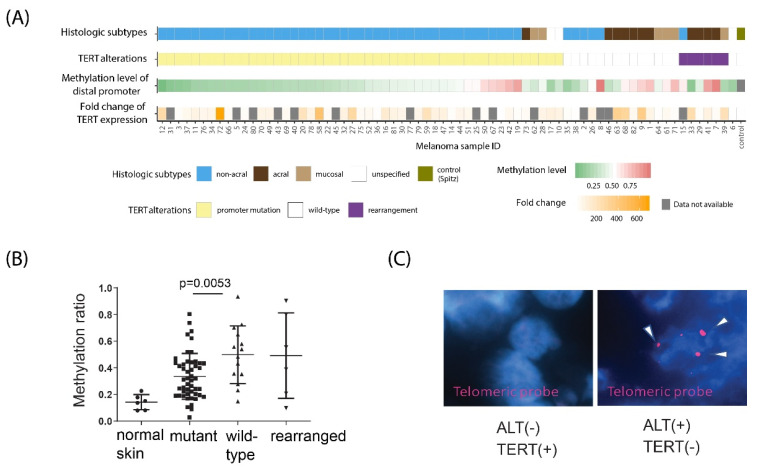
***TERT* promoter mutations, rearrangements, and promoter methylation are common in TERT–expressing adult metastatic melanomas**. (**A**) *TERT* promoter mutations, rearrangements, and promoter methylation levels in adult metastatic melanomas were assessed by Sanger sequencing, FISH, and high–throughput bisulfite sequencing, respectively. Histological subtypes of melanomas were determined by morphologic criteria in the primary tumors. TERT mRNA expression levels were measured by RT–qPCR and normalized to both control Spitz sample and GAPDH mRNA levels. Normal skin samples were used as negative controls for TERT expression. (**B**) Average methylation of the *TERT* distal promoter CpGs (chr5:1,295,586–1,295,771) in normal skin and in mutant, wild–type, and *TERT*–rearranged melanomas. (**C**) Telomere FISH signal collected with a Cy3–labeled telomere probe revealed that a *TERT* alteration–negative melanoma (sample #6) had exceptionally high telomere content (arrows; right panel), which is indicative of ALT mechanism activation. In comparison, a *TERT*–positive melanoma (left panel) did not exhibit a strong telomere FISH signal, consistent with no ALT-activation.

All melanomas, except for one (sample #6), harbored at least one of the three cancer-associated *TERT* alterations: promoter point mutation, distal promoter methylation, or rearrangement (Figure 1A and Supplemental Table S1). One melanoma that lacked any *TERT* alteration had exceptionally long telomeres, characteristic of ALT-mediated telomere maintenance (Figure 1C). In total, 49 of 70 melanomas (70%) harbored a *TERT* promoter mutation, including 1,295,228 C>T (*n* = 18), 1,295,250 C>T (*n* = 25), 1,295,242-43 CC>TT (*n* = 5), and 1,295,161 A>C (*n* = 1). A total of 6 of 70 melanomas (9%) had *TERT* rearrangement as assessed by FISH (Supplemental Figure S1), and 14 of 70 melanomas (20%) were wild type. Promoter mutations were completely mutually exclusive to structural rearrangements, but distal promoter methylation was not mutually exclusive to either mutations or rearrangements (Figure 1A). *TERT* wild-type melanomas were generally more methylated than mutant melanomas at the distal *TERT* promoter (one side Wilcox rank sum test, *p* = 0.0053; Figure 1B), but these aberrations were not mutually exclusive. By RT-qPCR (reverse transcription quantitative real-time PCR), all tested melanomas, except for one, expressed TERT, to various levels, while normal skin controls were negative for TERT expression (Figure 1A and Supplemental Table S2). As expected, the ALT-positive metastatic melanoma (sample #6) did not express TERT [2].

### 3.2. Active, Mutant TERT Promoter Alleles Are Less Methylated Than Their Silent, Mutation-Free Homologs in Heterozygous Mutant Cancer Cell Lines

**Figure 2:**
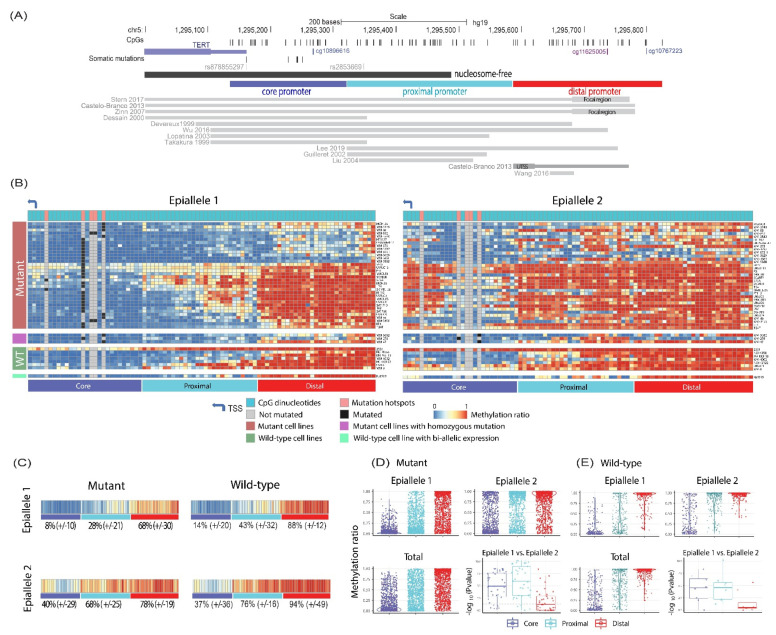
**Allelic methylation levels in***TERT***promoter mutant and wild-type cancer cell lines.** (**A**) *TERT* promoter map shows CpG dinucleotides, somatic point mutations, and SNPs in the 763 bp region (chr5:1,295,104–1,295,866, hg19). The start codon is located at chr5:1,295,104. Based on methylation profiles, three promoter regions were defined in this study: the core promoter ((187 bp, chr5:1,295,135–1,295,321), encompassing the recurrent promoter mutation sites), the proximal promoter (264 bp, chr5: 1,295,322–1,295,585), and the frequently methylated distal promoter region (239 bp, chr5: 1,295,586–1,295,824). The previously studied regions are denoted by the publication [20,24,28,35,36,37,38,39,40,41,42,43] (Supplemental Table S6). The thicker bars represent focal regions interrogated in a greater depth in those studies. (**B**) Methylation levels per CpG dinucleotides per epiallele in the *TERT* promoter. Epialleles of the *TERT* promoter were determined by iteratively clustering the CpG methylation patterns of the reads generated from high-throughput sequencing of bisulfite converted DNA amplification. Only the cell lines with two putative epialleles are shown (wild–type cell line *n* = 8; mutant cell line *n* = 37). (**C**) Averaged epiallele CpG methylation level in wild–type and mutant cell line reads. Average methylation values (+/− standard deviation) in the core, proximal, and distal promoter regions are indicated. (**D**) Distribution of the methylation ratio in the *TERT* core, the proximal, and the distal promoter regions is shown for epialleles in mutant cell lines. Each dot represents a CpG site in a cell line. In most of the examined cell lines, epiallele 2 has a significantly higher methylation ratio than epiallele 1 in both the core (85%, *n* = 29) and the proximal promoter (91%, *n* = 31) (*p* < 0.05, one–tail Wilcoxon rank sum test). (**E**) CpG methylation ratios were determined in wild-type cell lines as described for panel D. In all examined wild-type cell lines, except for bi-allelically expressing Ku1919 cell line (*n* = 7), epiallele 2 has a significantly higher methylation ratio than epiallele 1 in both the core and the proximal promoter region (*p* < 0.05, one–tail Wilcoxon rank sum test).

Given that some metastatic melanomas harbored both mutated and methylated *TERT* distal promoters, we next asked whether mutation and methylation tended to occur on the same promoter molecule (i.e., *in cis* on the same allele) or on separate molecules (i.e., *in trans* on homologous alleles). Because FFPE samples have lower tumor purity, due to admixture of normal cells, and yield more fragmented DNA than cancer cell lines, we focused on cancer cell lines to answer this question. We performed single-molecule high-throughput bisulfite amplicon sequencing in 36 heterozygous and 5 homozygous *TERT* promoter mutant cell lines, derived from melanoma and other cancers (e.g., urothelial, lung, and brain). We could differentiate active and silent allele-derived methylation reads (i.e., epialleles) for each cell line’s *TERT* promoter because we obtained a single long contiguous bisulfite converted amplicon DNA (Supplemental Figure S2A). This amplicon spanned from the distal promoter region to the core promoter, encompassing the mutation sites (Figure 2A). The predicted active/silent status of the promoter was significantly associated with the mean percentage of methylation in our analysis (logistic regression, odds ratio: 1.06, 95% CI: 1.03–1.08, *p* < 0.0001). In agreement with the previous reports [23,24,33,34], mutant alleles were significantly less methylated than their silent, mutation-free homologs in the heterozygous mutant cell lines (Figure 2; *p* < 0.05, one side Wilcox rank sum test). Our Chromatin Immunoprecipitation (ChIP) data were also consistent with the previous observations [24] that the mutant alleles are enriched in the active histone mark H3K4me2/3, while the mutation-free alleles are enriched in the repressive histone mark H3K27me3 (Supplemental Figures S2C–E and S3B).

### 3.3. TERT Distal Promoter Methylation and TERT Point Mutations Can Co-Occur on the Same Active TERT Promoter Alleles

Previous studies have shown that *TERT* promoter methylation and mutation tend to mark separate homologous alleles in heterozygous *TERT* mutant cell lines [23,24,33,34]. The mutual exclusivity of mutations and methylation, however, did not strongly hold when we compared the methylation levels of the active and silent alleles specifically at the *TERT* distal promoter; 50% (*n* = 17) of the mutant cell lines had no significant difference in the methylation level at the distal promoter between the active (epiallele 1) and silent (epiallele 2) alleles (Figure 2B). Only the core and proximal promoter were significantly less methylated in the active, mutant alleles compared to their silent, mutation-free alleles (Figure 2D; *p* < 0.05, one-tail Wilcoxon rank sum test). These results indicate that *TERT* distal promoter methylation may occur on the transcribed mutant alleles, and that only the core and proximal methylation opposes transcription (Figure 2B–D).

### 3.4. Transcriptionally Active TERT Promoter Alleles in Wild-Type Cancer Cell Lines Are Simultaneously Hypermethylated in the Distal Promoter and Hypomethylated in the Core and Proximal Promoter

**Figure 3:**
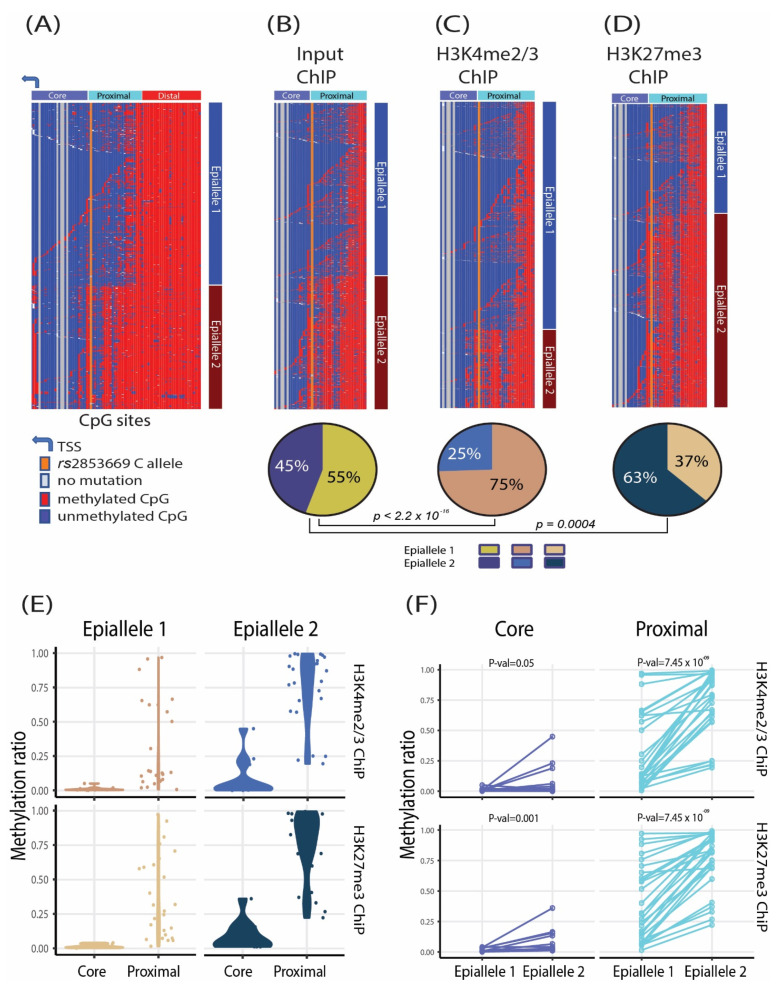
**The mono-allelically expressing wild-type NCI–H2122 cancer cell line shows distinct allele–specific *TERT* promoter methylation patterns**. (**A**) Methylation status of each CpG island per read in the *TERT* promoter (chr5:1,295,135–1,295,824) of wild–type NCI–H2122 is shown. The methylation status of the reads was clustered into two epialleles based on the breadth and depth of methylation. (**B**–**D**) Classification of epiallele 1 (less methylated) and epiallele 2 (more methylated) in NCI-H2122 promoters for (**B**) ChIP input sample DNA, (**C**) DNA enriched in the active histone mark H3K4me2/3 ChIP, and (**D**) DNA enriched in the repressive histone mark H3K27me3 ChIP. ChIP DNA was bisulfite converted, followed by the *TERT* promoter high-throughput targeted amplicon sequencing. Proportions of the read classified as epialleles 1 and 2 are shown as pie–charts. (**E**) Distribution of the methylation ratio in the *TERT* core and proximal promoter regions is shown for epialleles 1 and 2. Each dot represents a CpG site. The less methylated epiallele 1 is enriched for ChIP DNA with the active histone mark, whereas the more methylated epiallele 2 is enriched for ChIP DNA with the repressive histone mark. (**F**) Comparison of the methylation ratio of epialleles in the core and proximal regions. Epiallele 2 has a significantly higher methylation ratio than epiallele 1 in the proximal promoter (*p* = 7.45 × 10^−9^, one–tail Wilcox signed rank test).

Similar to the clinical melanoma samples, the wild-type cancer cell lines, on average, had significantly higher *TERT* distal promoter CpG methylation than mutant cell lines (*p* < 0.05, one-tail Wilcoxon rank sum test; Supplemental Figure S4). The mono- versus bi-allelic expression status was known for five wild-type cell lines (Supplemental Table S2); all, except for the Ku1919 cell line (Figure 2B), mono-allelically expressed *TERT*. We therefore looked for methylation patterns associated with active versus silent alleles, like in the heterozygous mutant cell lines. For each wild-type cell line, we clustered the methylation reads into two groups based on the breadth and depth of methylation (see Methods) and identified two distinct methylation patterns (epialleles 1 and 2) for eight wild-type cell lines (Figure 2B). In all cell lines, we found that both epialleles were symmetrically methylated to high levels in the *TERT* distal promoter (Figure 2B). By contrast, one epiallele was significantly more hypomethylated than the other in the *TERT* core and proximal promoter (*p* < 0.05 one-tail Wilcoxon rank sum test; Figure 2E). The wild-type cancer cell line methylation reads could not as easily be separated into active/silent allelic groups because these reads did not harbor informative promoter mutations that could serve as transcription status markers. To assign active or silent alleles, we performed ChIP to determine the distribution of histone marks on the respective epialleles. The integrated ChIP data showed that promoters with hypomethylated *TERT* core and proximal promoters were enriched in the H3K4me2/3 histone mark (i.e., active allele), whereas those with increased core and proximal methylation were enriched in the H3K27me3 histone mark (i.e., silent allele) (Figure 3B–F and Supplemental Figure S3C). Thus, in mono-allelically expressing wild-type cancer cell lines, high methylation levels of the *TERT* distal promoter associated not only with the silent alleles, but also with the active ones. The active alleles, however, retained low methylation levels at the core and proximal sites, in agreement with a previous report [44]. Both promoter alleles of the Ku1919 cell line exhibited the active promoter pattern (i.e., methylated distal promoter and hypomethylated core and proximal promoters), which is concordant with the bi-allelic expression of *TERT* in this cell line (Figure 2B). Like wild-type cell line reads, *TERT*-rearranged cell line reads could not be assigned to active or silent alleles based on mutation status but were instead segregated into transcriptionally undefined groups based on methylation profile (Supplemental Tables S1 and S2).

### 3.5. Decitabine Treatment Induces TERT Bi-Allelic Expression in Wild-Type Cancer Cell Lines

**Figure 4:**
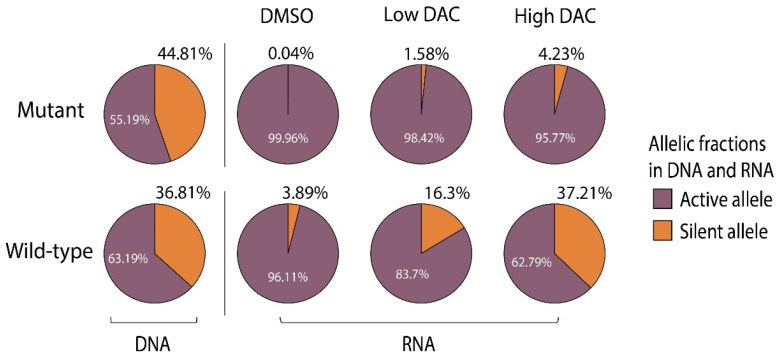
**Decitabine treatment induces***TERT***bi–allelic expression in mono-allelically expressing wild–type cancer cell lines.** Relative *TERT* allele-specific expression measured in four mutant and four wild-type cell lines treated with either DMSO or decitabine. Exonic SNP rs2736098 or rs2853690 allele ratios were quantified by ddPCR from cDNA using allele discriminatory TaqMan probes (ThermoFisher; Waltham, MA). cDNA was prepared from cells treated with either no decitabine (DMSO) or with decitabine (DAC) concentrations an order of magnitude lower (low [DAC]) or higher (high [DAC]) than the half maximal growth rate–inducing DAC concentration. DAC–concentration was titrated to empirically determine low and high [DAC] values for each cell line (Supplemental Table S3). Allele–specific expression was calculated by normalizing the relative cDNA prevalence of each allele with its relative genomic DNA prevalence, shown as allelic fractions in DNA and RNA. Results show biological replicates for four cell lines and triplicates for two cell lines, but also include a single experiment for the mutant cell lines VMCUB3 and WM–88. Relative expression values of less–expressed alleles are given.

To test if disruption of the highly methylated *TERT* distal promoter would perturb TERT expression, we treated four mono-allelically expressing wild-type cell lines and seven genetically altered cancer cell lines for comparison (five with point mutations and two with rearrangement). We measured transcription and methylation changes of each allele upon treatment. Decitabine treatment reactivated silent allele transcription in all wild-type cell lines (i.e., induced bi-allelic expression; Figure 4, bottom panel). In contrast, decitabine did not induce bi-allelic expression in the genetically altered cell lines (Figure 4, top and middle panel). To our surprise, we did not observe any change in the *TERT* distal promoter methylation in these cell lines. However, decitabine treatment reduced the *TERT* core and proximal promoter methylation in the wild-type cell lines (Supplemental Figure S5 and Supplemental Table S3).

Bi-allelic expression was not due to decitabine-induced toxicity [45] because even low decitabine concentrations induced bi-allelic expression (see Methods) [46]. Decitabine treatment increased H19 RNA levels in both wild-type and genetically altered cell lines, which served as a positive control for drug efficacy (Supplemental Figure S6) [47,48]. Overall, these results indicate that the DNA demethylating agent activates silent *TERT* alleles in wild-type but not in genetically altered cell lines and suggest that wild-type transcriptional re-activation may be associated with demethylation of the *TERT* core and proximal promoter region upon decitabine treatment.

## 4. Discussion

The long-read methylation sequencing of cancer cell lines revealed previously unknown differences in *TERT* methylation pattern and demethylating agent responses between wild-type and genetically altered cancer cell lines. In mutant cancer cell lines, robust methylation and mutation were more mutually exclusive; the silent, mutation-free allele was more methylated than the transcriptionally active, mutated allele. In the wild-type cancer cell lines, by contrast, both alleles (regardless of the transcriptional state) were methylated to similarly high levels in the *TERT* distal promoter. Finally, decitabine treatment reactivated silent alleles in wild-type cell lines but not genetically altered cell lines.

Our cancer cell line analysis was different in several ways from many previous *TERT* promoter methylation and expression studies. First, because *TERT* is often regulated at the allele level [5,29], and in most cancer cells not all *TERT* alleles are transcribed [5,25], we looked at the two major allelic methylation patterns from each cell line separately, instead of averaging them together [24,37]. This prevented masking of the methylation characteristics of the active allele due to mixing with those of the silent allele. Second, we examined promoter methylation patterns by bisulfite sequencing of one contiguous amplicon, instead of stitching sequences of shorter non-overlapping amplicons together [23,24,33]. This allowed us to compare methylation levels in the core, proximal, and distal promoters of the active and silent homologs across a long, single DNA molecule. Third, we searched for both *TERT* promoter point mutations and *TERT* structural rearrangements to identify *TERT* genetically altered samples; sometimes promoter mutations are taken into account while rearrangements are overlooked. Finally, we measured decitabine’s effect on TERT allele-specific expression levels instead of total TERT mRNA expression levels [20,29,36,37,49,50,51]. This approach revealed previously unappreciated findings in the methylation profiles and responses to demethylating agents between wild-type and genetically altered cancer cell lines.

Much of the previous interest in *TERT* allele-specific expression and promoter methylation in cancer has focused more on *TERT* mutant samples [23,24]. Allele-specific promoter methylation and allele-specific expression have not been systematically and comprehensively studied in wild-type cancers [25]. Our single-molecule analysis in mutant cancer cells confirmed previous findings that the transcriptionally silent, mutation-free allele is more heavily methylated than the transcriptionally active, mutant allele [24,33]. This supports the idea of promoter DNA methylation acting as a transcriptional repressor [24,34]. We hence predicted that the bulk of methylation in wild-type cancers should similarly reside in the transcriptionally silent alleles. Surprisingly, we found that both alleles in wild-type cancer cell lines (including the bi-allelically expressed Ku1919 cell line) had symmetrically high methylation levels at the *TERT* distal promoter. These findings suggested that methylation of the *TERT* distal promoter may be functionally compatible, or even functionally important, especially for TERT wild-type cancers. We therefore investigated whether methylation disruption perturbed TERT expression in wild-type cell lines. We found that decitabine treatment could reactivate the silent alleles in wild-type cell lines but not in genetically altered ones. In tandem with the transcriptional reactivation of the silent alleles, the methylation levels of the *TERT* core and proximal promoter dropped, whereas those of the distal promoter remained unchanged.

Like promoters of all actively transcribed alleles, the transcribed *TERT* promoters of all cancer cell lines, regardless of the mutational status, were hypomethylated in the core and proximal promoter. This hypomethylated area is probably synonymous with the *TERT* nucleosome-free region around the upstream of the TSS [10,52,53,54,55] (Figure 2A and Supplemental Tables S3–S5). It is known that some wild-type cancer cell lines display mono-allelic TERT expression and others bi-allelic expression [5,24], but it is unclear what determines mono- or bi-allelic expression in these cases. We propose that wild-type mono-allelic expression may result from random allelic silencing by nucleosome-free region methylation during in vitro propagation of some cell lines [56,57,58]. Decitabine treatment restored bi-allelic expression, indicating that wild-type cell line allelic silencing is not irreversible. We hypothesize that the nascent silent allele was transformed into an active one by decitabine-induced demethylation in the nucleosome-free region that overlapped the *TERT* core and proximal promoter regions. Methylation of the nucleosome-free region is incompatible with transcription [52,53,59,60], and decitabine-induced demethylation of the core and proximal promoter may allow transcription-factor-mediated nucleosome displacement. This is also supported by previous reports showing that promoter demethylation can temporarily reactivate epigenetically silenced tumor suppressor genes [61].

Despite these new observations, it is still unclear whether wild-type *TERT* distal promoter methylation is a bona fide activating mechanism equivalent to the mechanistically established genetic alterations, because decitabine treatment did not significantly change the distal promoter methylation in these experiments. It is also conceivable that decitabine’s impact on global demethylation could indirectly affect the expression of TERT. Further studies are needed to examine the transcriptional effects of methylation in wild-type cancers by specifically targeting different regions of the *TERT* promoter.

Our methods have some inherent limitations: (1) Analysis of clinical samples was useful in demonstrating that *TERT* promoter alterations are ubiquitous in metastatic melanoma. Almost every metastatic melanoma in our cohort harbored one of three cancer-associated *TERT* aberrations (promoter point mutation, structural rearrangement, or methylation). Although *TERT* promoter methylation was more prevalent in melanomas that contained wild-type *TERT* promoter, our data showed that methylation and mutation or rearrangement were not mutually exclusive at the sample level. However, targeted long-read bisulfite sequencing was not feasible with these FFPE clinical specimens to gather more comprehensive data, because their DNA is more fragmented than intact cell line DNA. (2) A potential complication of the current long-read approach could be the formation of PCR chimeras during the amplification stage prior to sequencing, which could result in chimeric reads and false allele group prediction [44]. (3) The clustering step of methylation for long reads data was limited to 4. If the number of allele status is >2, the allele status is undetermined for groups displaying transitional patterns.

## 5. Conclusions

The heterogeneity of allele-specific *TERT* promoter methylation profiles of *TERT* wild-type and mutant cancer cells had remained unclear, particularly how they vary in the core, proximal, and frequently methylated distal promoter regions, despite having been substantially studied for more than two decades (Figure 2A). The single-molecule methylation analysis used in our study allowed us to reveal methylation differences between wild-type and mutant cancer cell lines. Homologous alleles in wild-type cancer cell lines, unlike the genetically altered ones, were symmetrically methylated at high levels in the *TERT* distal promoter. The *TERT* proximal and core promoter methylation, however, was different in each case; these regions were less hypomethylated in the silent alleles than the transcribed counterparts. Additionally, the wild-type and genetically altered cell lines responded differently to the demethylating agent decitabine. The nascent allele was reactivated by demethylating agent treatment in wild-type cell lines but not in genetically altered cell lines. These findings suggest that *TERT* allele-specific expression in vitro may be epigenetically regulated in wild-type cancer cell lines, in contrast to the genetically altered cells. Finally, the long-read based methylation profiling methods used for comparing allelic methylation patterns and allele-specific expression may be applicable to other genes where promoter methylation correlates with expression. This approach allows future fine mapping of the exact allelic specific regulatory elements in the promoter regions by integrating other epigenetic modifications including histone marks, chromatin opening, as well as transcription factor binding assays.

## Data Availability

Not applicable.

## Supplementary Materials

The following supporting information can be downloaded at: https://www.mdpi.com/article/10.3390/cancers14164018/s1, Figure S1: Three melanomas and two cancer cell lines with TERT rearrangements detected by interphase break-apart FISH. (A) A melanoma harboring the 1,295,250 C>T mutation (left) that lacked a TERT rearrangement was used as a negative control. (B) TERT rearrangements were detected by FISH in glioblastoma (LN-18) and melanoma (WM-51) cancer cell lines. A melanoma cell line (WM-88) harboring the 1,295,250 C>T mutation (left) that lacked a TERT rearrangement was used as a negative control. TERT rearrangements were seen using bacmid CH17-75N21- (red) and CH17-410B01- (green) templated probes, which correspond to regions upstream and downstream of the TERT gene, respectively. Figure S2: The TERT promoter mutant allele is associated with an active histone mark in the heterozygous mutant cancer cell line UMUC3, while the mutation-free allele is associated with a repressive histone mark. (A) The allele-specific TERT promoter methylation patterns in the mutant UMUC3 urothelial cancer cell line. gDNA was subjected to bisulfate treatment, PCR amplification, and high-throughput targeted amplicon sequencing. The proportion of 1,295,228 C>T mutant and wild-type alleles were quantified in the cell line DNA. TERT promoter methylation patterns were determined in the region of chr5:1,295,135–1,295,824 (hg19). Mutant (active) and mutation-free (silent) read groups/alleles were defined based on the presence or absence of the 1,295,228 C>T mutation, respectively. Red and blue indicate methylated and unmethylated CpGs, respectively. (B) Quantification of UMUC3 mutant (active) and mutation-free (silent) promoter methylation levels for each amplicon CpG. ChIP assay shows (C) in ChIP input DNA (D) enriched in DNA with the active histone mark H3K4me2/3 ChIP and (E) enriched in DNA with the repressive histone mark H3K27me3 ChIP. Allele proportions were quantified by ddPCR using TERT promoter mutation allele discrimination TaqMan probes. Figure S3: Hypomethylated allele of the TERT promoter is associated with an active histone mark, while hypermethylated allele is associated with a silent histone mark both in the heterozygous mutant cancer cell lines and the mono-allelically expressing wild-type cancer cell lines. (A) TERT promoter positions (chr5: 1,295,224–1,295,546) of the core and proximal region for ChIP analysis. Sanger sequencing of bisulfite PCR amplicon for ChIP DNA input, DNA enriched in the active histone mark H3K4me2/3 ChIP, and DNA enriched in the repressive histone mark H3K27me3 ChIP in (B) A375 and UMUC3 mutant cancer cell lines and in (C) 253J and NCI-H358 wild-type cancer cell lines. Sanger sequencing of bisulfite converted, PCR amplified ChIP DNA was performed. Relative proportions were shown by ChIP DNA as methylated CpG indicated by blue and bisulfite-converted T (non-methylated CpG) indicated by red in arrow positions. DNA enriched in the repressive histone mark H3K27me3 ChIP shows a higher methylation Sanger sequencing peak than DNA enriched in the active histone mark H3K4me2/3 ChIP in the TERT core and proximal promoter both in the heterozygous mutant and wild-type cancer cell lines with mono-allelic expression. Figure S4: Wild-type cancer cell lines have significantly higher methylation levels than mutant cancer cell lines in the TERT distal promoter. CpG methylation ratios were compared in the core (top), proximal (middle), and distal promoter (bottom) regions between wild-type (*n* = 8) and mutant cancer cell lines (*n* = 37) on epiallele 1 (A) and epiallele 2 (B). The wild-type cell lines had higher CpG methylation levels than mutant cell lines in the distal promoter (*p* < 0.05, one-tail Wilcoxon rank sum test and t-test). Figure S5: Decitabine (DAC) treatment induces TERT core and proximal promoter demethylation in wild-type cancer cell lines. For each cell line, the average core (A), proximal (B), and distal promoter (C) CpG methylation was calculated on both epialleles in DMSO- and DAC-treated samples. Median fold changes in methylation levels of DAC-treated samples relative to DMSO-treated wild-type (*n* = 3) and mutant (*n* = 5) cell lines are shown. DAC-treated sample methylation values were normalized to DMSO-treated sample values. (D) TERT promoter methylation patterns in DMSO- or DAC-treated heterozygous mutant cell lines (UMUC3 and WM-46) and wild-type cancer cell lines (253J and WM-4002). gDNA was prepared from cells treated with either DMSO or high DAC concentration, as in Figure 4. The CpG methylation of the reads was generated from high-throughput sequencing of bisulfite-converted DNA amplicons. TERT promoter methylation patterns were determined in the region of chr5:1,295,135–1,295,824 (hg19). Mutant (active) and mutation-free (silent) read groups/epialleles 1 and 2 were defined based on the presence or absence of the promoter mutation, respectively. Red and blue indicate methylated and unmethylated CpGs, respectively. Given the increased heterogeneity of methylation patterns in the 253J cell line, the epialleles were defined purely according to the methylation profiles of the long reads. Figure S6: Decitabine treatment affects H19 RNA expression in cancer cell lines. (A) H19 RNA levels were measured in DMSO or DAC-treated wild-type cell lines by RT-qPCR. TATA-binding protein mRNA levels were used for normalization. cDNA was prepared from cells treated with either DMSO or DAC, as in Figure 4. Results of one experiment are shown. (B) H19 RNA levels in DMSO or DAC-treated mutant (WM-88, UMUC3, and WM-46) and rearranged (WM-51) cell lines were measured, as in panel A. Results of two (UMUC3 and WM-88) or one (all other cell lines) biological replicate experiments are shown. Three technical replicate measurements were made for each sample. Error bars indicate standard deviation. Table S1: Cancer cell lines (*TERT* promoter mutation, methylation and rearrangement assessment). Table S2: Melanoma histology, *TERT* promoter mutation, methylation and rearrangment assessement and TERT expression measurements. Table S3: DMSO- and DAC-treated cancer cell line *TERT* promoter methylation measurements. Table S4: Cancer cell lines TERT allele-specific expression (ASE) measurements. Table S5: DMSO- and DAC-treated cancer cell lines TERT allele-specific expression (ASE) measurements. Table S6: Genomic location (hg19) of *TERT* promoter characterized in the literatures and CpG sites used in this study.
