# Supplementary material for: Targeted Long-Read Bisulfite Sequencing Identifies Differences in the TERT Promoter Methylation Profiles between TERT Wild-Type and TERT Mutant Cancer Cells
Source: Cancers (Basel). 2022 Aug 19;14(16):4018. doi: 10.3390/cancers14164018 (PMC9406525; doi:10.3390/cancers14164018)

Supplemental Figure 3

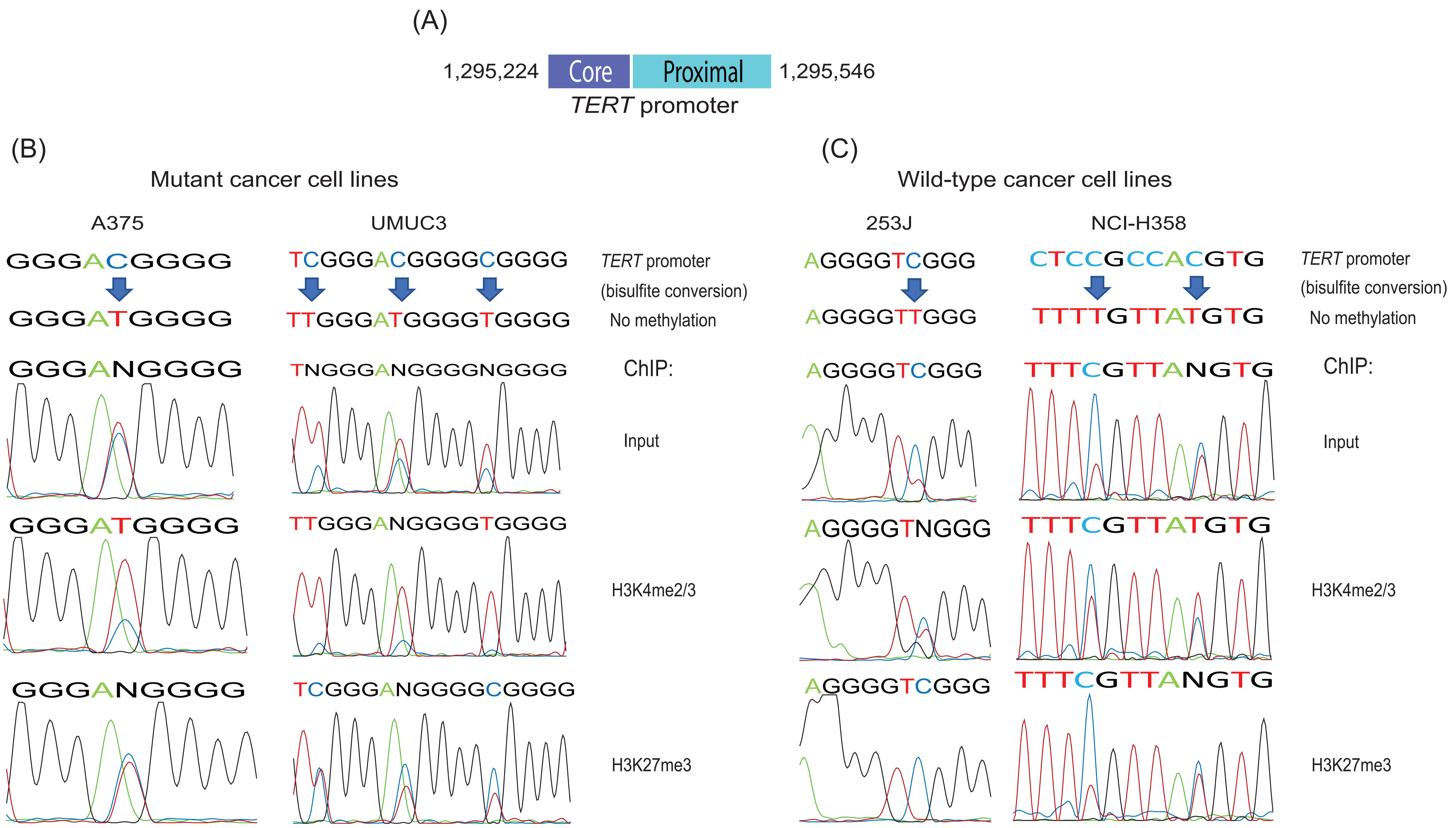

Supplement: Supplementary file 1 [file cancers-14-04018-s001.zip › SupplementaryMaterials/Supplemental Figure 3.pdf]
